# SOCIAL BRIDGING NETWORKS AND COGNITIVE AGING AMONG OLDER ADULTS: ORIGINS IN SOCIAL CONTEXTS, ROLES, AND ACTIVITIES

**DOI:** 10.1093/geroni/igad104.3724

**Published:** 2023-12-21

**Authors:** Brea Perry, Siyun Peng, Anne Krendl

**Affiliations:** Indiana University, Bloomington, Indiana, United States; Indiana University, Bloomington, Indiana, United States; Indiana University, Bloomington, Indiana, United States

## Abstract

There is a strong link between social connectedness and cognitive aging, with recent research pointing to social bridging being a primary underlying mechanism. Social bridging may provide cognitive enrichment through interactions with expansive, heterogeneous, and loosely interconnected social networks of weaker, peripheral ties. Drawing on egocentric social network data and cognitive assessments of 436 older adults from the Social Networks and Alzheimer’s Disease (SNAD) study, this paper explores where social bridging networks come from. We used multivariate regression to examine whether a latent social bridging factor comprised of six structural variables (network size, density, effective size, diversity, strength of weakest tie, and sole bridging status) was associated with cognitive function and social conditions. Results indicate that social bridging is significantly associated with global cognitive function (B= 0.19; p<.0001), verbal memory (B=0.26; p<.0001), and episodic memory (B=0.21; p<.0001), consistent with prior studies. Older adults who are women (b=0.40; p<.001) and have more years of education (b=0.05; p<.05) have more access to social bridging. Among community and housing characteristics, only perceived community integration is significantly correlated with higher bridging (b=0.22; p<.05). With respect to social roles, being currently married (b=0.39; p<.001) and being a volunteer (b=0.58; p<.0001) were positively associated with bridging, while being a veteran predicted lower bridging (b=-0.86; p<.0001). Among indicators of social engagement, only number of contacts in one’s phone (in 100’s) was associated with bridging (b=.16; p<.0001). Findings provide insight into social roles and activities that can be leveraged to expand bridging and improve cognitive aging outcomes.

